# Factors Related to Nurses' Professional Autonomy When Caring for Patients with COVID-19 in a University Hospital: A Cross-Sectional Study

**DOI:** 10.1155/2023/1741721

**Published:** 2023-10-11

**Authors:** Sambuu Ganchuluun, Akiko Kondo, Megumi Ukuda, Hiroki Hirai, Jieru Wen

**Affiliations:** ^1^International Nursing Development, Graduate School of Health Care Sciences, Tokyo Medical and Dental University, Tokyo, Japan; ^2^Mongolia-Japan Hospital, Mongolian National University of Medical Sciences, Ulaanbaatar, Mongolia; ^3^Tokyo Medical and Dental University Hospital, Tokyo, Japan

## Abstract

Although individual factors play a vital role in determining professional autonomy, their specific impact during the coronavirus disease 2019 (COVID-19) pandemic has not been studied. This study aimed to compare nurses' professional autonomy when caring for patients with COVID-19 and for other patients and explore factors related to autonomy when caring for these patients. A paper-based questionnaire survey was conducted from June to August 2022 among nurses working at a university hospital in Japan. The survey included demographic factors (10 items, including, age, section, years of nursing experience, position, educational background, ladder level, and having certified nurse specialists or certified nurse qualifications) and individual experience factors (4 items: number of COVID-19 patients experienced, frequency and contents of searching for the latest information about COVID-19, frequency of using scientific sources, and frequency of training/study sessions on COVID-19 attended at the hospital). Additionally, basic knowledge of COVID-19 was evaluated. The scale for nurses' professional autonomy was developed based on a previous study. A paired *t*-test and stepwise multiple linear regression were used for the analyses. Overall, 241 nurses participated in the survey. The average length of nursing experience was 10.3 ± 9.2 years. The total scores for nurses' professional autonomy in all 5 factors 27 items were significantly lower (*t* = −12.1, *p* < 0.001) when caring for COVID-19 patients than when caring for other patients. Specifically, Factor 1 (Cognition) exhibited the most decreased scores when caring for COVID-19 patients than when caring for other patients. Factor 4 (Abstract judgment) differed the least between caring for COVID-19 and for other patients, but the average score was the lowest. More years of nursing experience (*β* = 0.208, *p* = 0.001) and a higher number of patients with COVID-19 cared for (*β* = 0.140, *p* = 0.026) were associated with higher autonomy scores. In conclusion, to enhance professional autonomy during an unprecedented pandemic, nurses must enhance cognition and abstract judgment. In the event of a future pandemic, nurses need to create an environment in which they routinely access and utilize the latest information and scientific evidence to provide high-quality nursing care based on their professional judgment and competence.

## 1. Introduction

The coronavirus disease 2019 (COVID-19) was caused by the severe acute respiratory syndrome coronavirus 2 (SARS-CoV-2) and became a global pandemic [1]. The first cases of novel coronavirus were detected in China in December 2019, and the virus has then spread rapidly to other countries worldwide. This led the World Health Organization to declare a Public Health Emergency of International Concern on January 30, 2020, and to characterize the outbreak as a pandemic on March 11, 2020 [1]. As of March 16, 2023, over 760 million confirmed cases and over 6.8 million deaths have been reported globally since the COVID-19 pandemic started, with a large number of patients requiring medical treatment and intensive care in hospitals [1, 2]. However, this phenomenon occurs not only in hospitals but also in clinics, home nursing service stations, midwifery centers, and other facilities [3]. The COVID-19 pandemic has required nurses to provide quality nursing care for patients with COVID-19 even though they are not trained for this unprecedented situation.

Nurses' professional autonomy is defined as “having the authority to make decisions and freedom to act in accordance with one's professional knowledge” [4]. There are two main components of nurses' professional autonomy: independence in decision-making and the ability to utilize one's own competence. Nursing competence is defined as the ability of a nurse to effectively demonstrate a set of attributes, such as personal characteristics, values, attitudes, knowledge, and skills that are required for nurses to fulfill their professional responsibilities [5]. The utilization of personal competence has been recognized as necessary for nurses' autonomy, including deciding their approach to nursing care [6]. Nurses' professional autonomy is strongly related to job satisfaction, job stress, and psychological distress, which can affect their work engagement [7–10].

Factors that affect nurses' professional autonomy reported before COVID-19 can be classified into individual and environmental factors. Individual factors including sex, educational level, nursing experience, job position, critical thinking, professional skills, and clinical ladder level contribute to the utilization of nurses' professional autonomy [11, 12]. Male nurses have higher independence in decision-making than female nurses. Educational level, nursing experience, job position, critical thinking, and clinical ladder are strongly and positively associated with nurses' professional autonomy [10–12]. Furthermore, the number of patients experienced is also related to nurses' professional autonomy [13]. Meanwhile, environmental factors that can affect nurses' professional autonomy include supportive leadership [14], shared leadership, interprofessional and intraprofessional collaboration, and healthy work environments [6]. Supportive leadership can enhance nurses' professional autonomy, as it has a significant impact on decision-making in nursing care and fosters a positive workplace environment. In contrast, autocratic/nonsupportive management may adversely affect nurses' autonomy [14]. Autocratic management is typically marked by a leader's inclination to centralize decision-making and consolidate power, often resulting in complete control over all aspects of their subordinates' activities, with little regard for input from the subordinates [15]. Nurse-physician collaboration and cooperation among nurses without authoritarian impositions could increase nurses' professional autonomy [16]. Meanwhile, poor nurse-physician relationships could reduce nurses' professional autonomy [14]. A healthy working environment promotes nurses' autonomy through good team spirit without conflict or teasing and a well-balanced workload [14].

The COVID-19 pandemic had a significant impact on nurses, with an increase in psychological distress and a decrease in job satisfaction [7, 17]. Nurses experienced significant stress and moral distress due to unfavorable job demands [17]. Common reasons for moral distress include a lack of decision-making autonomy, insufficient workplace respect, and inadequate psychosocial support mechanisms to help nurses cope with ethical dilemmas in both intensive care units [18] and general units [17, 19–21]. One study indicated that nurses' psychological stress during the COVID-19 pandemic could be related to their lower autonomy and competence [7]. However, it is unclear how the current pandemic has affected nurses' professional autonomy. Previous studies have investigated the relationship between environmental factors and professional autonomy during the pandemic. One study reported that the nurse-physician relationship improved because of the COVID-19 pandemic, and shared education and collaboration had a significantly positive influence on the nurses' professional autonomy [16]. Other studies reported that a high level of autonomy was associated with greater organizational citizenship behavior [22, 23] and low work-family conflict [24]. Organizational citizenship behavior is a type of cooperative behavior that increases a person's tendency toward helping and sharing information [22, 23]. However, no study has reported individual factors that can predict professional autonomy during the COVID-19 pandemic. One study reported that more younger nurses felt their professional autonomy increased than older nurses during the pandemic [25], but this study did not measure and compare professional autonomy using scores. Therefore, it was unknown whether age is related to increased autonomy.

Although individual factors play a vital role in determining professional autonomy, their specific impact in this particular scenario has not been studied. It is necessary to identify the individual factors that can affect professional autonomy during a pandemic, which can be used to develop individual training and educational programs. Therefore, this study specifically focused on investigating individual factors. This study aimed to (1) compare nurses' professional autonomy of the same nurses when caring for patients with COVID-19 and other patients and (2) explore individual factors related to nurses' autonomy when caring for patients with COVID-19.

## 2. Materials and Methods

### 2.1. Study Design

This cross-sectional study used the convenience sampling method as researchers' accessibility to participants. This study was approved by the Ethics Review Committee of the Institute of Education of Tokyo Medical and Dental University (Approval No.: C2022-01) and was conducted according to the tenets of the Declaration of Helsinki. Informed consent was obtained from all participants.

### 2.2. Participants and Setting

Participants were nurses who were employed at the 750-bed university hospital in an urban area of Japan during the study period. The study hospital had been actively contributing to measures against COVID-19 since 2020. The inclusion criteria were working at the hospital for more than 1 year and having experience caring for COVID-19 patients. Nurses in the director's office were excluded. A paper-and-pencil questionnaire was provided to nurses. Data were collected between July and August 2022.

### 2.3. Data Collection

The first author explained the purpose, significance, and methods of the survey to the nurse managers of each section at their meeting after approval from the director of nursing. The informed consent included the explanation about voluntary participation with no rewards or demerits regardless of participation, anonymity, and not to be used for work evaluation. The first author provided a total of 997 survey forms in the director of the nursing office, which were divided into 30 sections according to the number of nurses in each section. The nurse manager in each section took the survey forms to his/her section and asked nurses to voluntarily collaborate on the survey at their convenience and return it after putting it into the sealed envelope to the designated collection bags provided in their respective sections. Nurse managers returned the bags containing the questionnaires in sealed envelopes, as well as any remaining survey forms, in 2-3 weeks to the director of the nursing office, and the survey materials were collected by the first author.

### 2.4. Variables and Instruments

The survey consisted of four main parts: (1) demographic factors (10 items), (2) individual experience factors (4 items), (3) basic knowledge of COVID-19 (20 items), and (4) professional autonomy questions (27 items). Items included demographic factors, individual experience factors, and basic knowledge of COVID-19 which were individual factors potentially related to professional autonomy based on previous research.

Demographic factors included sex [12], age [12], section, years of nursing experience [7, 12, 13], position [11], educational background [12], ladder level [24], and having certified nurse specialists (master level) or certified nurse (six-months training) qualifications. Individual experience factors included the number of COVID-19 patients experienced [13], frequency and contents of searching for the latest information about COVID-19, frequency they used scientific sources [26], and frequency of training/study sessions on COVID-19 attended at the hospital [19]. Basic knowledge of COVID-19 [4, 7] included coronavirus disease, infection period, polymerase chain reaction (PCR) test, nasopharyngeal antigen test, risk of complications, and infection control measures. These individual experience factors and basic knowledge are considered to be related to professional autonomy [4, 6, 27].

The questions for knowledge about COVID-19 were developed by researchers, including a middle-level nurse caring for patients with COVID-19 and a nurse manager of the COVID-19 ward who was not included in the research team based on her ward's COVID-19 guidelines. The ward's COVID-19 guidelines were previously created by external experts, and the head nurse confirmed the contents. Participants responded right or wrong for each statement, and the number of correct answers was counted. The possible scores ranged from 0 (lowest) to 20 (highest). As the pilot test, five midlevel nurses with experience in caring for COVID-19 patients were asked to answer the 20 items for basic knowledge of COVID-19 twice with a 3-day interval [27]. We used the test-retest reliability to assess the total score stability with the intraclass correlation coefficient (ICC). The consistency was considered favorable if the ICC value was ≥0.70 [27], and the ICC in the current study was 0.833.

Contents of searching for the latest information about COVID-19 included general information about COVID-19 (the disease itself), the contagion of COVID-19, infection control measures, clinical studies results, treatment methods of COVID-19, nursing care for patients with COVID-19, nursing case study of the patients with COVID-19, others, and not searched. These items were also decided by researchers and the nurse manager of the COVID-19 ward based on the necessary knowledge to take care of patients with COVID-19 [4, 7, 26]. Participants were asked if they ever searched or not for each item.

Regarding nurses' professional autonomy, we modified the scale developed by Kikuchi and Harada published in 1997 after obtaining Kikuchi's permission [28]. Although there are several scales to measure nurses' professional autonomy, we selected the scale for the following reasons. One review [29] showed that 15 measures of nurses' autonomy were developed worldwide between 1974 and 2015, and 3 of them were developed by Japanese researchers. The scale developed by Kikuchi [28] was used in more nursing studies (eight) in Japan and had higher internal consistency than the scale developed by Tao in 1979 and the professional autonomy scale in nursing developed by Shijiki in 1999 [30]. Overall, 7 of the 12 professional autonomy scales outside of Japan are commonly used [29]. Among these 7 scales, 3 are validated in Japanese [31–33]. However, these scales may not necessarily be appropriate for the Japanese situation, including items such as “I consider I will gain the proper education and experience and begin working independently like a nurse practitioner” because there is no nurse practitioner who can work independently in Japan.

Kikuchi viewed nursing activities as cognition, judgment, and performance and developed their scale for Japanese nurses. Kikuchi original scale included 5 subscales with 47 items, namely, cognition (14 items), performance (14 items), concrete judgment (7 items), abstract judgment (7 items), and independent judgment (5 items) [28]. This scale covers the two main components of nurses' professional autonomy [6]: independence in decision-making including three types of judgments and the ability to utilize own competence of cognition and performance. During the COVID-19 pandemic, nurses were required to make decisions by themselves and work in unprecedented situations. Therefore, these scales were important in our study, which was conducted during the COVID-19 pandemic. However, Kikuchi's original scale was developed more than two decades ago and has some items that were not necessary in our study. Thus, researchers including the supervising professor discussed with the head nurse of the COVID-19 ward and modified it to fit the current clinical situation. The number of items was reduced to 27 items to decrease the participant burden. The revised scale included cognition (9 items), performance (6 items), concrete judgment (4 items), abstract judgment (6 items), and basic nursing judgment (2 items). Each item was rated on the same 5-point Likert scale, with the scores ranging from 1 (strongly disagree) to 5 (strongly agree). The total score ranged from 27 to 135. Pilot testing using the revised scale was conducted among the same five middle-level nurses with nursing experience caring for COVID-19 patients, and face validity was confirmed. The internal consistency of the pilot study for the total score of nurses' professional autonomy when caring for COVID-19 patients was evaluated using Cronbach's *α*, with a value of ≥0.7 considered acceptable [34]. Cronbach's *α* in this was 0.952. We also used test-retest reliability to assess the score stability, with an ICC of 0.963 for the total score. The ICC values of the 5 factors were 0.960, 0.905, 0.860, 0.900, and 0.885, respectively.

### 2.5. Statistical Analyses

The target sample size for this study was calculated using G^*∗*^Power (version 3.1.9.7). To detect a small effect size (d) of 0.2 with an *α* error of 0.05 (two-tailed) and a power of 0.8 by the paired *t*-test, 199 participants were required. Participant characteristics are described as descriptive statistics. The normality of data distribution was assessed using the skewness-kurtosis test, with a skewness of < −2 or >2 defined to indicate non-normally distributed data [35]. The total scores of the autonomy scale when caring for COVID-19 patients had a skewness value of −0.22 and a kurtosis value of 1.61; when caring for other patients, the values were −0.83 and 1.34, respectively. Therefore, the data were regarded to be normally distributed; hence, parametric tests were used. A paired *t*-test was used to compare the total score, average score of each factor, and each autonomy score between caring for COVID-19 patients and for other patients. The statistical significance level was set at *p* < 0.01 to decrease alpha error in the paired *t*-test for 27 individual items. Cohen's d was used to compare the effect size of the difference between caring for COVID-19 patients and for other patients among the factors and items of professional autonomy.

The total scores of professional autonomies when caring for COVID-19 patients were compared among three or more groups (sections) using an analysis of variance. Further, comparisons between two independent groups such as sex, age (20–40 years vs. 41–60 years), having a qualification of certified nurse specialist/certified nurse or not, and searching for the latest information about COVID-19 or not were performed using an independent *t*-test. Spearman's correlation coefficients (*ρ*) were used to analyze the association between nurses' professional autonomy when caring for patients with COVID-19 and non-normally distributed continuous variables such as age (four groups), years of nursing experience, and ordinal variables of educational level, position, ladder level, frequency of searching for the latest information about COVID-19, frequency of using scientific sources, number of COVID-19 patients cared for, and frequency of attending training/study sessions at the hospital. Significant factors related to nurses' autonomy when caring for COVID-19 patients in the bivariate analyses (*p* < 0.1) were included in the stepwise multiple linear regression analysis to explore the independent factors related to nurses' professional autonomy. The normality of the residuals was confirmed using the Durbin–Watson ratio (between 1.5 and 2.5) [36]. Multicollinearity was checked using the variance inflation factor (VIF) (<10.0). An alpha level of 0.05 was set to indicate significance in the stepwise multiple linear regression. All statistical analyses were performed using SPSS version 22 (SPSS Inc., Chicago, IL, USA) software.

## 3. Results

### 3.1. Participant Demographics and Experiences

A total of 318 questionnaires were collected, yielding a response rate of 31.8%. Duplicate responses were checked using identified duplicate cases function in SPSS, and no duplicates were identified. Overall, 63 participants were excluded because they had not cared for COVID-19 patients. After excluding 14 questionnaires with missing values, data from 241 participants were used in the analysis (effective response rate, 94.5%).  Table 1 presents the participants' characteristics. The average length of nursing experience was 10.3 ± 9.2 years. In total, 51.9% of the respondents were aged ≤30 years, and 93.4% were female. Most participants were staff nurses (87.6%) and had a Bachelor's degree (70.1%), with only 1.2% having a Master's degree. The most frequent educational backgrounds by age group were as follows: 92.0% of nurses aged 20–30 years and 76.8% of those aged 31–40 years completed a 4-year university nursing program, and 51.4% of those aged 41–50 years and 80% of those aged 51–60 years completed a 3 year vocational nursing school program. For the distribution according to station, 20.7% of nurses were from internal medicine; 30.7%, surgery; 11.6%, outpatient; and 36.9%, other stations. Over half (59.3%) of the participants did not attend any training sessions on COVID-19 at the hospital during the pandemic (Table 1). Approximately, 60% of the participants searched the information about COVID-19 (the disease itself), while less than 5% of them searched for the results of clinical studies and nursing case studies about patients with COVID-19 (Table 2).

### 3.2. Basic Knowledge of COVID-19

The average number of correct answers was 17.4 ± 1.4.  Table 3 shows the questions ranked in order by the highest correct rates. Five items, such as the fatality rate of COVID-19 and utilization of N95 masks when caring for infectious patients, were responded to 100% correctly (nos. 1–5). Eight items, including risk factors of severe disease, personal protective equipment (PPE) usage when working with a deceased body, choosing rapid PCR testing for patients suspected of COVID-19, surgical mask and N95 using situations, PPE usage when performing environmental disinfection, and infectious period of COVID-19 (nos. 6–13), had a correct response rate of over 90%. However, 4 items (nos. 17–20), including the peak viral shedding and the incubation period of COVID-19 and practices (alcohol disinfection and sanitizing patients' dishes) for infection prevention and control, had a low correct response rate (<70%). The item with the lowest correct response rate (33.6%) pertained to the order of removing PPE (no. 20).

### 3.3. Differences in Autonomy Scores between Caring for COVID-19 and Other Patients

The total score for nurses' professional autonomy was significantly lower (*t* = −12.1, *p* < 0.001, Cohen's *d* = 0.780) when caring for COVID-19 patients than for other patients (Table 4). The scores for the five factors and all their items were significantly lower when caring for patients with COVID-19 than for other patients (*p* < 0.001). The highest mean score when caring for COVID-19 patients was for Factor 2 (performance), while the lowest mean score was for Factor 4 (abstract judgment). The highest Cohen's d was for Factor 1 (cognition), while the lowest Cohen's d was for Factor 5 (basic nursing judgment). Cohen's d of two items in Factor 1 was ≥0.7: “I can predict the physical effects of the treatment on the patient” and “I can predict future problems that may occur to the patient based on the course of events to date.” Further, 7 items had Cohen's d of 0.6 to 0.5, and 18 items had Cohen's d of 0.4 to 0.3. The item with the lowest Cohen's d (0.314) was “I can use the latest scientific evidence including the nursing articles to decide appropriate nursing,” which belonged to Factor 4 (Abstract judgment). Table 4 includes the overall Cronbach's alphas and the subscales for both caring situations.

### 3.4. Factors Influencing Nurses' Professional Autonomy When Caring for Patients with COVID-19

Table 1 shows the association between the total score for professional autonomy when caring for patients with COVID-19 and each variable. Participants aged ≥41 years had higher professional autonomy than those aged ≤40 years (*p*=0.015). Years of nursing experience (*ρ* = 0.163, *p*=0.011), number of COVID-19 patients experienced (*ρ* = 0.176, *p*=0.006), and frequency of attending training/study sessions about COVID-19 at the hospital (*ρ* = 0.150, *p*=0.020) were positively associated with professional autonomy. Although clinical ladder was positively associated with professional autonomy (*ρ* = 0.152, *p*=0.018), the highest professional autonomy (96.0 ± 21.0) was observed among nurses with a clinical ladder level “0.”

The total score of professional autonomy did not significantly differ according to four age groups, sex, stations, position, nursing certification, educational background, frequency, and contents searched for the latest information about COVID-19, and frequency of using scientific sources.

Based on the bivariate analyses, 2 age groups (20–40 and 41–60 years), years of nursing experience, clinical ladder level, frequency of attending training/study sessions at the hospital, and the number of COVID-19 patients cared for were entered into the stepwise multiple linear regression analysis. The results indicated that longer years of nursing experience (*β* = 0.208, *p*=0.001, adjusted *R*^2^ = 0.040) and a higher number of patients with COVID-19 experienced (*β* = 0.140, *p*=0.026, adjusted *R*^2^ = 0.015) were significantly related to higher autonomy scores. The explanatory variance of these two factors was 5.5% (Table 5).

## 4. Discussion

This study found that nurses' professional autonomy with all investigated five factors and 27 items was significantly lower when caring for COVID-19 patients than when caring for other patients. Specifically, Factor 1 (cognition) exhibited the most decreased scores when caring for COVID-19 patients than when caring for other patients. Factor 4 (abstract judgment) showed the smallest difference between caring for COVID-19 and for other patients, but the score was the lowest. On the other hand, Factor 5 (basic nursing Judgment) demonstrated relatively higher scores, even when caring for COVID-19 patients. Factors related to higher professional autonomy when caring for COVID-19 patients were longer years of nursing experience and experiencing more patients with COVID-19. To the best of our knowledge, this is the first study to compare the professional autonomy of the same nurses when caring for COVID-19 and for other patients and to report individual factors related to professional autonomy when caring for COVID-19 patients using a scale.

Among the five factors of professional autonomy, Factor 1 (cognition) exhibited the highest Cohen's d score, with 5 items greater than 0.5. The predictive items for patient outcomes were significantly lower when caring for COVID-19 patients than when caring for other patients. The items with the highest Cohen's d scores were to predict the effects of the treatment and future problems, to identify changes in a patient's condition, and to understand the relationship between test results and symptoms. These results may be attributed to a lack of sufficient information on COVID-19, limited experience in acquiring the necessary knowledge and skills to predict treatment responses, and provision of appropriate care for COVID-19 patients [37], resulting in an inability to effectively apply knowledge, skills, and judgment [5].

Factor 5 (basic nursing judgment) had the lowest Cohen's d, and the average score was relatively high with the items focused on choosing appropriate nursing methods considering a patient's needs. It is assumed that nurses gained the basic nursing skills required to work with COVID-19 patients, which can also be common with other patients. Additionally, the item about using the latest scientific evidence in nursing practice in Factor 4 (abstract judgment) showed the smallest difference between caring for COVID-19 and for other patients, but the average score was the lowest. Although it was important for the participants to obtain appropriate information about the unprecedented COVID-19 pandemic, very few participants searched for clinical studies and nursing practice cases, consistent with previous reports [38–40]. Collectively, these results support that most nurses may not use scientific evidence in their nursing practice, regardless of the current pandemic situation [38–40]. In Japan, approximately 12% of nurses working in hospitals had a university degree, and 1.3% had completed graduate education [41]. In our study, although the majority of the participants were university graduates (68.9%), only a similar proportion held Master's degree (1.2%). This indicates that undergraduate education alone is inadequate to apply scientific evidence and achieve higher professional autonomy in pandemic situations.

Further, in our study, age, educational background, clinical ladder level, and using scientific sources were not related to nurses' professional autonomy when caring for COVID-19 patients. A previous study reported that years of experience, but not age, was significantly correlated to the nurses' professional autonomy [8], consistent with the current results. Regarding educational background, three nurses had Master's degrees and had the highest professional autonomy score in this study. Despite having higher educational backgrounds, young nurses (i.e., those aged ≤40 years) had fewer years of nursing experience and tended to exhibit lower levels of professional autonomy compared with older nurses (i.e., those aged ≥41 years). Additionally, it should be noted that some older nurses who did not take the clinical ladder were on level 0 despite their longer nursing experience and higher professional autonomy. In our study, less than 5% of the participants searched for results of clinical studies and nursing case studies about patients with COVID-19. There was no obvious association between using scientific sources and professional nursing autonomy, although previous studies reported that using scientific sources increased critical thinking [26] and that critical thinking is strongly associated with professional nursing autonomy [26]. Previous studies reported that educational backgrounds were significantly related to the use of research results [42–46], in which over 15% of nurses had a Master's degree. In our study, only 1.2% of nurses had a Master's degree, which can be the reason that the educational levels were not significantly related to professional autonomy scores.

Basic knowledge of COVID-19 and attending training/study sessions on COVID-19 at the hospital were not related to nurses' professional autonomy, either. The correct response rates regarding the peak viral shedding and the incubation period of COVID-19 items (nos. 16–17) were lower than those of the standard precaution items of infection prevention and control (nos. 2–5), which were common infection control protocols and not specific for COVID-19. The item with the lowest correct response rate (33.6%) was the order of PPE removal. PPE removal is a complicated multistep task that requires not only knowledge but also multiple practice. A previous study reported no significant correlation between knowledge and nurse compliance with the use of level 2 PPE [47], and consistent findings were observed in the current study. This indicates that experience-based learning systems such as simulation and role play may be necessary for improving nurses' professional autonomy, in addition to acquiring knowledge.

### 4.1. Limitations

This current study has several limitations. First, the findings cannot be generalized to all hospital settings because the study was conducted at a university hospital in an urban area in Japan, where the educational level is relatively high. Nationwide surveys, not only in Japan but also in other countries, need to be conducted. Second, subjectivity and response bias are inherent challenges in self-reported questionnaires. The response rate (31.8%) was not high enough, which could have caused a participation bias, in which nurses with higher professional autonomy could have been included. Third, because there were no rewards for participation and no duplicated answers were found, it was unlikely that the same person responded more than once; however, there is no guarantee that this was the case in this anonymous study. Fourth, the timings of working with COVID-19 patients or other patients were not specified, and some nurses might not have been working with other patients at the time of the investigation. Participants had to consider their previous experiences, which could have caused a memory bias. Fifth, the current study reported a low model fit (*R*^2^ = 0.055); only approximately 6% of the variance related to professional autonomy was explained. More factors related to professional autonomy other than those measured in this study should be explored. As this study focused on individual, rather than environmental factors, it is also necessary to further investigate the environmental factors that may affect professional autonomy during the pandemic. Finally, it is not possible to establish causality among variables owing to the cross-sectional study design.

Regardless of these limitations, our study contributes by expanding the existing body of research and offering novel insights into nurses' autonomy. Although factors related to professional autonomy during the COVID-19 pandemic were similar to those before the pandemic, our findings indicate that “cognition” is vulnerable to decrease and “abstract judgment” is as low as during usual conditions among professional autonomy domains.

### 4.2. Practical Implications

To enhance professional autonomy during unprecedented pandemics, nurses must enhance cognition and abstract judgment. To enhance cognition and abstract judgment, it is necessary to promote the integration of up-to-date information and clinical evidence into their practice, and the following recommendations at the three levels are suggested.

At the individual level, for nurses to be able to predict patients' condition and mitigate the uncertainty experienced during the COVID-19 pandemic, it is crucial to actively obtain information about patients' progress. Nurses should attend the information-sharing meetings and training.

At the organizational level, it is necessary to guide nurses on how to access up-to-date clinical information and research evidence and effectively utilize it. Creating study groups sharing clinical cases and experiences among multiple professionals is crucial in fostering collaboration and knowledge exchange, which may promote nurses' professional growth and autonomy, empowering them to deliver high-quality care. Healthcare organizations should also incorporate experience-based simulations and role-play practice into their training programs to allow nurses to practice and apply their skills in realistic scenarios. Hospital administrators also need to hire more nurses with graduate education, who play a role in connecting research and practice.

At the policy-making level, it is necessary to make accurate up-to-date information about the infectious disease available to healthcare professionals as soon as possible. In the long run, it is necessary to mandate more education in undergraduate nursing programs on how to use research evidence and to encourage and support more nurses to study in graduate programs. In addition, support may be needed for universities to strengthen research and measures to develop experience-based simulations, such as using virtual reality technology. Further studies are required to clarify the effects of experience-based learning systems in enhancing professional autonomy.

## 5. Conclusions

Nurses' professional autonomy in all 5 factors and 27 items was significantly lower when caring for COVID-19 patients than when caring for other patients. Specifically, Factor 1 (cognition) exhibited the most decreased scores when caring for COVID-19 patients than when caring for other patients. Factor 4 (abstract judgment) showed the smallest difference between them, but the average score was the lowest. Factors related to higher professional autonomy when caring for COVID-19 patients were longer years of nursing experience and experiencing more patients with COVID-19. To enhance professional autonomy during an unprecedented pandemic, it is necessary for nurses to enhance cognition and abstract judgment. In the event of a future pandemic, nurses need to create an environment in which they routinely access and utilize the latest information and scientific evidence so that they can provide high-quality nursing care based on their own professional judgment and competence. Experience-based learning systems may also be necessary to enhance professional autonomy.

## Figures and Tables

**Table 1:** Participant characteristics and association with the total score of professional autonomy when caring for patients with COVID-19 (*n* = 241).

Characteristics	Categories	*n* (%)/M ± SD	Average professional autonomy score ± SD when caring for patients with COVID-19	*ρ* ^a^	*p*
Age, years (four groups)	20–30	125 (51.9)	90.1 ± 13.6	0.100	0.122^a^
	31–40	56 (23.2)	89.2 ± 15.8		
	41–50	35 (14.5)	92.7 ± 12.1		
	51–60	25 (10.4)	98.4 ± 16.9		

Age, years (two groups)	20–40	181 (75.1)	89.8 ± 14.3		0.015^b^
	41–60	60 (24.9)	95.1 ± 14.4		

Sex	Male	16 (6.6)	93.3 ± 6.7		0.534^b^
	Female	226 (93.4)	91.0 ± 14.9		

Years of nursing experience		10.3 ± 9.2		0.163	0.011^a^

Position	Staff nurse	211 (87.6)	90.6 ± 14.5	0.093	0.152^a^
	Assistant nurse manager	23 (9.5)	93.4 ± 11.4		
	Nurse manager	7 (2.9)	100.7 ± 19.6		

Educational background	High school nursing course, 5 years	7 (2.9)	97.0 ± 15.2	−0.096	0.136^a^
	Vocational nursing school, 3 years	51 (21.2)	95.1 ± 14.2		
	Junior college of nursing, 3 years	14 (5.8)	89.6 ± 13.9		
	University nursing course, 4 years	166 (68.9)	89.5 ± 14.2		
	Graduate school (Masters of Science)	3 (1.2)	106.0 ± 23.0		

Stations	Internal medicine	50 (20.7)	88.9 ± 14.3		0.618^c^
	Surgery	74 (30.7)	91.4 ± 11.4		
	Outpatient	28 (11.6)	90.9 ± 15.9		
	Other (including COVID-19 ward)	89 (36.9)	92.3 ± 16.4		

Clinical ladder level	0	17 (7.1)	96.0 ± 21.0	0.152	0.018^a^
	I	38 (15.8)	84.7 ± 12.5		
	II	66 (27.4)	90.3 ± 12.9		
	II	59 (24.5)	92.7 ± 15.7		
	IV	45 (18.7)	92.5 ± 12.2		
	V	16 (6.6)	95.5 ± 14.7		

Having a certified nurse specialist or certified nurse qualifications	Yes	11 (4.6)	95.2 ± 12.8		0.341^b^
	No	231 (95.4)	90.9 ± 14.6		

Number of COVID-19 patients experienced	1–10	103 (42.7)	88.3 ± 14.0	0.176	0.006^a^
	11–20	30 (12.4)	95.4 ± 13.6		
	21–30	16 (6.6)	88.1 ± 16.2		
	31–40	9 (3.7)	98.8 ± 12.6		
	41–50	13 (5.4)	95.1 ± 11.9		
	51–100	22 (9.1)	92.4 ± 11.5		
	≥101	37 (15.4)	93.8 ± 17.4		
	Missing data	11 (4.6)			

Frequency of using scientific sources	Never	77 (32.0)	89.4 ± 14.8	0.073	0.260^a^
	Once a year	12 (5.0)	90.3 ± 15.5		
	Once every 6 months	32 (13.3)	92.3 ± 12.7		
	Once every 3 months	31 (12.9)	90.2 ± 13.9		
	Once a month	42 (17.4)	92.7 ± 12.0		
	Once every 1–2 weeks	32 (13.3)	92.0 ± 19.0		
	More than once every 1–2 weeks	15 (6.2)	94.3 ± 13.9		

Frequency of searching for latest information about COVID-19	Never	18 (7.5)	87.3 ± 14.4	0.082	0.203^a^
	Once a year	4 (1.7)	93.2 ± 17.3		
	Once every 6 months	16 (6.6)	91.4 ± 13.3		
	Once every 3 months	28 (11.6)	87.7 ± 19.2		
	Once a month	58 (24.1)	91.6 ± 10.8		
	Once every 1–2 weeks	79 (32.8)	91.3 ± 12.9		
	More than once every 1–2 weeks	38 (15.8)	94.2 ± 19.4		

Frequency of attending training/study sessions about COVID-19 at the hospital	Never	143 (59.3)	89.1 ± 13.8	0.150	0.020^a^
	Once a year	55 (22.8)	95.1 ± 12.3		
	Once every 6 months	30 (12.4)	92.6 ± 18.3		
	Once every 3 months	8 (3.3)	99.3 ± 18.2		
	Once a month	4 (1.7)	82.7 ± 14.1		
	More than once every 1–2 weeks	1 (0.4)	84.0 ± 0.0		

Total score of the basic knowledge of COVID-19		17.4 ± 1.4		−0.014	0.831^a^

^a^The associations between the total score of nurses' professional autonomy when caring for patients with COVID-19 and ordinal and continuous variables are assessed using Spearman's correlation coefficients. ^b^Independent *t*-test. ^c^Analysis of variance.

**Table 2:** Overview of recent information search on COVID-19 (*n* = 241).

	Factors	*n*	%
1	About COVID-19 (disease itself)	144	20.5
2	Spread situation of the infection	180	25.6
3	Infection control measures	136	19.3
4	Results of clinical studies	25	3.6
5	Treatment methods	105	14.9
6	How to care for patients with COVID-19	65	9.2
7	Nursing case studies about patients with COVID-19	27	3.8
8	Others	11	1.6
9	Not searched	11	1.6
	Total	241	100

**Table 3:** Basic knowledge of COVID-19 (*n* = 241).

	Items	Correct response (%)
1	The fatality rate remains constant regardless of age or underlying diseases	100.0
2	Wear an N95 mask when suctioning airway secretions from suspected or positive patients	100.0
3	Wear an N95 mask while performing endotracheal intubation on suspected or positive patients	100.0
4	Wear an N95 mask when applying noninvasive positive pressure ventilation (NPPV) to suspected or positive patients	100.0
5	Wear an N95 mask when performing bronchoscopy on suspected or positive patients	100.0
6	Chronic kidney disease, type 2 diabetes, and hypertension are not risk factors for severe disease	99.6
7	Personal protective equipment is not required when providing direct care to the dead body of an infected patient	99.2
8	If there is a suspicion of COVID-19, even if the antigen test was negative, one rapid PCR testing should be performed in the hospital	98.8
9	A surgical mask is sufficient when talking to suspected or positive patients at a distance	98.3
10	Wear an N95 mask when performing cardiopulmonary resuscitation (CPR) on suspected or positive patients	97.5
11	If the patient (positive) is not present, the staff member performing the environmental disinfection is not required to wear gloves and gowns	97.1
12	If you are only serving meals to suspected or positive patients in their hospital rooms, only surgical masks and gloves are required	96.3
13	The infectious period starts 2 days prior and lasts 7–10 days after the onset of symptoms	95.4
14	If a new COVID-19 infection is suspected after admission, even if the screening test result is negative, a nasopharyngeal antigen test should be performed promptly	87.1
15	Respiratory therapy options for critically ill patients are high-flow oxygen therapy and noninvasive positive pressure ventilation	71.8
16	The peak of viral shedding for COVID-19 is 2-3 days after onset	70.5
17	The incubation period of COVID-19 (omicron strain) is approximately 2-3 days	69.7
18	Alcohol is effective as a disinfectant against COVID-19, but hypochlorous acid water is not	66.0
19	Always wash and sanitize dishes used by patients (positive) separately from other patients	58.5
20	Order of removing personal protective equipment (PPE), in case of double gloving: hand disinfection ⟶ outer gloves ⟶ hand disinfection ⟶ remove gown and inner gloves while turning them inside out ⟶ hand disinfection ⟶ face shield and goggles ⟶ hand disinfection ⟶ cap ⟶ hand disinfection ⟶ mask ⟶ hand disinfection	33.6

**Table 4:** Differences in nurses' professional autonomy scores between caring for COVID-19 and other patients (*n* = 241).

Items	When caring for COVID-19 patients		When caring for other patients		Cohen's d	*p*
	Mean ± SD	*α*	Mean ± SD	*α*		
Factor 1: cognition	3.4 ± 0.5	0.85	3.8 ± 0.4	0.87	0.86	<0.001
I can predict the physical effects of the treatment on the patient	3.3 ± 0.8		3.9 ± 0.5		0.72	<0.001
I can predict future problems that may occur to the patient based on the course of events to date	3.2 ± 0.8		3.8 ± 0.6		0.70	<0.001
I can accurately identify changes in a patient's general condition	3.2 ± 0.8		3.6 ± 0.6		0.64	<0.001
I can understand the relationship between a patient's test results and symptoms	3.4 ± 0.7		3.8 ± 0.5		0.60	<0.001
I can fully understand the values of my patients	3.2 ± 0.8		3.5 ± 0.7		0.57	<0.001
I can understand the discrepancy between a patient's verbal and emotional behavior	3.3 ± 0.8		3.6 ± 0.6		0.48	<0.001
I can predict the psychological impact of hospitalization and treatment on the patient	3.5 ± 0.8		3.9 ± 0.6		0.47	<0.001
I am able to gather the information needed for nursing care (patient's personality, lifestyle, psychological problems he/she may have)	3.5 ± 0.8		3.9 ± 0.5		0.46	<0.001
I can show empathic understanding of the patient's words and actions	3.7 ± 0.8		4.0 ± 0.6		0.43	<0.001

Factor 2: performance	3.5 ± 0.6	0.85	3.8 ± 0.5	0.84	0.62	<0.001
I am able to provide nursing care based on the patient's distinctive characteristic of individual or social life	3.4 ± 0.7		3.8 ± 0.5		0.53	<0.001
I am able to prioritize and systematically carry out my nursing duties	3.7 ± 0.6		3.9 ± 0.5		0.52	<0.001
I can cooperate with other professionals	3.7 ± 0.7		4.2 ± 0.6		0.48	<0.001
I can allay a patient's distrust and anxiety about medical care by providing sufficient explanation	3.3 ± 0.7		3.6 ± 0.6		0.46	<0.001
I am able to provide nursing care calmly in an emergency	3.1 ± 0.9		3.4 ± 0.8		0.45	<0.001
I can handle a patient's emotions (anger, sadness)	3.4 ± 0.7		3.7 ± 0.6		0.38	<0.001

Factor 3: concrete judgment	3.4 ± 0.7	0.83	3.8 ± 0.6	0.85	0.61	<0.001
I can assess a patient's condition from various information and choose nursing care that is appropriate for the patient's needs	3.3 ± 0.7		3.7 ± 0.6		0.57	<0.001
I can perform nursing assessments and provide recommendations to achieve the highest quality of care	3.4 ± 0.7		3.8 ± 0.6		0.50	<0.001
I can respond to sudden physiological changes in the patient (hematemesis, loss of consciousness, hypotension, chills)	3.3 ± 0.9		3.6 ± 0.7		0.46	<0.001
I can prioritize a patient's problem and clarify the most important problem among the many problems	3.5 ± 0.7		3.7 ± 0.6		0.42	<0.001

Factor 4: abstract judgment	3.2 ± 0.7	0.89	3.4 ± 0.6	0.86	0.50	<0.001
I can proactively present patient's problems and lead to solve it at the medical meetings	3.2 ± 0.8		3.5 ± 0.7		0.46	<0.001
I can evaluate a patient's symptoms and test results and select appropriate nursing methods	3.4 ± 0.7		3.7 ± 0.6		0.45	<0.001
I can make nursing decisions based on the medical situation without sufficient information	2.9 ± 0.8		3.1 ± 0.9		0.39	<0.001
I can plan nursing care using the nursing model and introduce it to my coworkers	3.2 ± 0.8		3.4 ± 0.7		0.34	<0.001
The nursing plan that I developed can be approved by my coworkers	3.4 ± 0.7		3.6 ± 0.7		0.32	<0.001
I can use the latest scientific evidence including nursing articles to decide on appropriate nursing	2.7 ± 0.9		2.9 ± 1.0		0.31	<0.001

Factor 5: basic nursing judgment	3.5 ± 0.7	0.83	3.7 ± 0.6	0.75	0.43	<0.001
I can choose nursing methods considering the patient's needs	3.5 ± 0.7		3.8 ± 0.6		0.42	<0.001
I can choose appropriate nursing methods, regardless of the patient's words and actions	3.4 ± 0.7		3.6 ± 0.6		0.33	<0.001

Total scores	91.1 ± 14.5	0.95	99.9 ± 11.8	0.94	0.78	<0.001

Paired *t*-test, *α*: Cronbach's *α* coefficients. The items within each factor are arranged in descending order of Cohen's d.

**Table 5:** Factors related to nurses' professional autonomy when caring for patients with COVID-19 (*n* = 241).

Factors	*β*	*T*	*p*	VIF	Adjusted *R*^2^
Years of nursing experience	0.208	3.311	0.001	1.000	0.040
Number of patients with COVID-19 experienced	0.140	2.233	0.026	1.000	0.015

Stepwise multiple linear regression. Dependent variable: total scores of nurses' professional autonomy when caring for COVID-19 patients. Final model: Durbin–Watson = 2.115, overall adjusted *R*^2^ = 0.055. VIF: variance inflation factor of all variables.

## Data Availability

The data used to support the findings of this study are available from the corresponding author upon request.
